# Case report: Stenosis turned leak … and turned stenosis—complications of paravalvular prosthetic leak closure with a plug device

**DOI:** 10.3389/fcvm.2023.1132063

**Published:** 2023-06-12

**Authors:** Barbara Pitta Gros, Olivier Roux, Eric Eeckhout, Matthias Kirsch

**Affiliations:** ^1^ Department of Cardiology, Lausanne University Hospital, Lausanne, Switzerland; ^2^ University of Lausanne, Lausanne, Switzerland; ^3^ Department of Cardiac Surgery, Lausanne University Hospital, Lausanne, Switzerland

**Keywords:** percutaneous valve therapy, paravalvular leak repair, aortic valve disease percutaneous intervention, transcatheter aortic valve implantation (TAVI), case report

## Abstract

**Background:**

Paravalvular leak is one of the most common complications and is among the most important prognostic factors of short- and long-term mortality after transcatheter aortic valve implantation (TAVI). Percutaneous valvular leak repair constitutes a first-line treatment for paravalvular leaks and is associated with high success rates and few serious complications nowadays. To the best of our knowledge, this is the first case where placement of the device through the stenting of the bioprosthesis resulted in creating a new symptomatic stenosis that required surgery.

**Case summary:**

We present a case of a patient with low-flow, low-gradient aortic stenosis treated with transfemoral implantation of a biological aortic prosthesis. One month after the procedure, the patient presented with acute pulmonary oedema and a paravalvular leak was discovered, which was corrected by percutaneous repair with a plug device. Five weeks after the valvular leak repair, the patient was readmitted for heart failure. At this time, a new aortic stenosis and paravalvular leak were diagnosed and the patient was referred for surgery. The new aortic mixed diseased was caused by the positioning of the plug device through the valve's metal stenting, which resulted in a paravalvular leak and pressed against the valve's leaflets, causing valvular stenosis. The patient was referred for surgical replacement and evolved well afterward.

**Conclusion:**

This case illustrates a rare complication of a complex procedure, and it highlights the need for multidisciplinary decisions and good cooperation between the cardiology and cardiac surgery teams to develop better criteria in the selection of the appropriate technique for managing paravalvular leaks after TAVI.

## Introduction

Despite the broadening of indications for transcatheter aortic valve implantation (TAVI) to include low-risk patients, as supported by subsequent studies (1, 2), some complications still undermine the use of this technique. Paravalvular leak (PVL) is one of the most common complications and is amongst the most important prognostic factors of mortality at short- and long-term after TAVI (3–5), being associated with a threefold increase in 30-day mortality (95% CI: 1.73–5.02) and a 2.3-fold increase in 1-year mortality (95% CI: −1.84 to 2.81) for moderate to severe leaks (6).

When comparing surgical to percutaneous aortic valve replacement, the incidence of moderate to severe paravalvular leaks between the percutaneous and the surgical series did not differ significantly in the PARTNER trial: the percutaneous group presenting 0.6% and the surgical group 0.5% at 1 year. By contrast, mild paravalvular leak at 1 year is still significantly higher in the percutaneous series, with 29.4% compared to 2.1% in the surgical one (2).

Here, we present the first case of paravalvular leak after TAVI treated percutaneously with an *Amplatzer* device where migration of the device resulted in severe aortic stenosis needing a surgical intervention.

## Case presentation

A 79-year-old female with a history of hypertension, permanent atrial fibrillation, and progressing aortic stenosis presented with NYHA stage II dyspnoea and peripheral oedema. Echocardiography showed a tricuspid aortic valve with severe paradoxical low-flow, low-gradient aortic stenosis with a surface of 0.5 cm^2^ by planimetry, a mean gradient of 19 mmHg, a preserved ejection fraction at 60%, and a low left ventricular output of 25 ml/min/m^2^ due to moderate hypertrophy. A CT scan revealed a modified Agatston calcium score of 630, with a calcium volume of 197 mm^3^ and moderate calcifications of the valve with heterogenous peripheral distribution. The patient's surgical risk was characterised by an EuroSCORE II of 1.60% and an STS score of 3.4% of predicted mortality, and her frailty score was at class 5.

The case was discussed in a multidisciplinary Heart Team meeting, and due to the patient's persistent symptoms and recurring hospitalisations, despite the low calcium score, an invasive strategy was decided. The patient was strongly opposed to cardiac surgery, despite her relatively low surgical risk, which contributed to the decision to perform a percutaneous aortic valve implantation.

The patient underwent a transfemoral implantation of a biological aortic prosthesis type *Edwards Sapien 3* of 23 mm, with a good echocardiographic result, mild paravalvular leak, and a mean valve gradient that came down to 8 mmHg.

**Figure 1:**
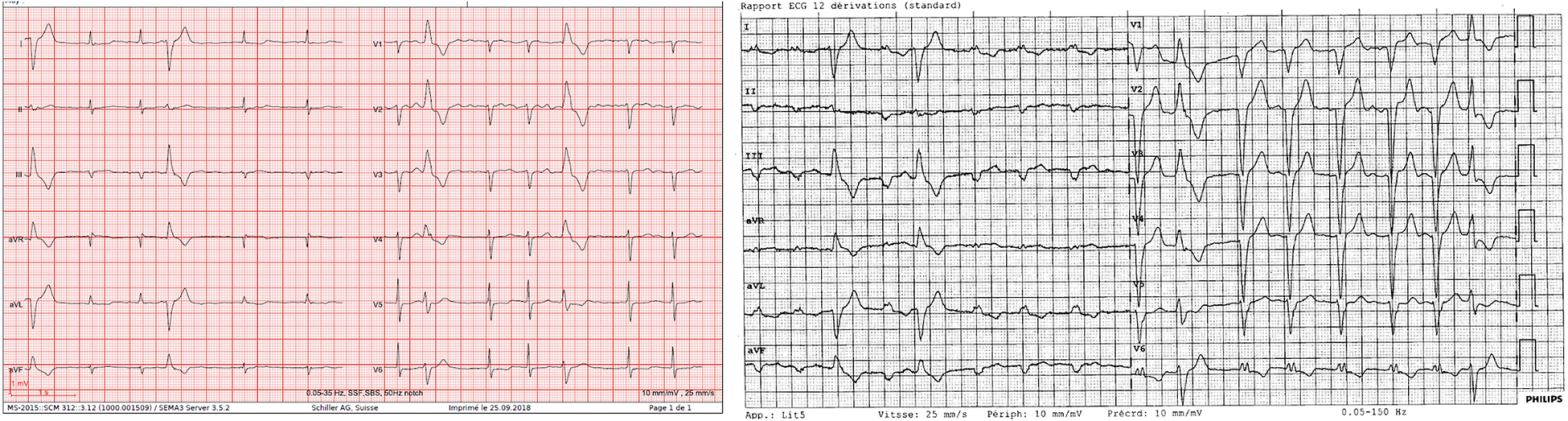
Left side: ECG before procedure showing atrial fibrillation, narrow QRS, and two premature ventricular contractions; right side: ECG after procedure showing a large QRS with a left bundle branch block.

Immediately after the procedure, a new left bundle branch block was noted (Figure 1) that motivated a His-ventricular (HV) exploration. A His-right ventricle conduction delay of 64 ms was found, which increased to 95 ms after Ajmaline provocation. Considering these results and given that the patient was in permanent atrial fibrillation with a difficult-to-control heart rate, despite bitherapy, the decision to implant a pacemaker was made. The patient was implanted with a single-chamber pacemaker SORIN, followed by an atrioventricular node ablation.

One month after the procedure, the patient presented with acute pulmonary oedema. Physical examination revealed a new diastolic heart murmur, and the echocardiography confirmed a seemingly significant paravalvular leak with an ERO (Effective Regurgitant Orifice) by PISA method at 0.1 cm^2^ and a planimetry of 0.4 cm^2^.

**Figure 2:**
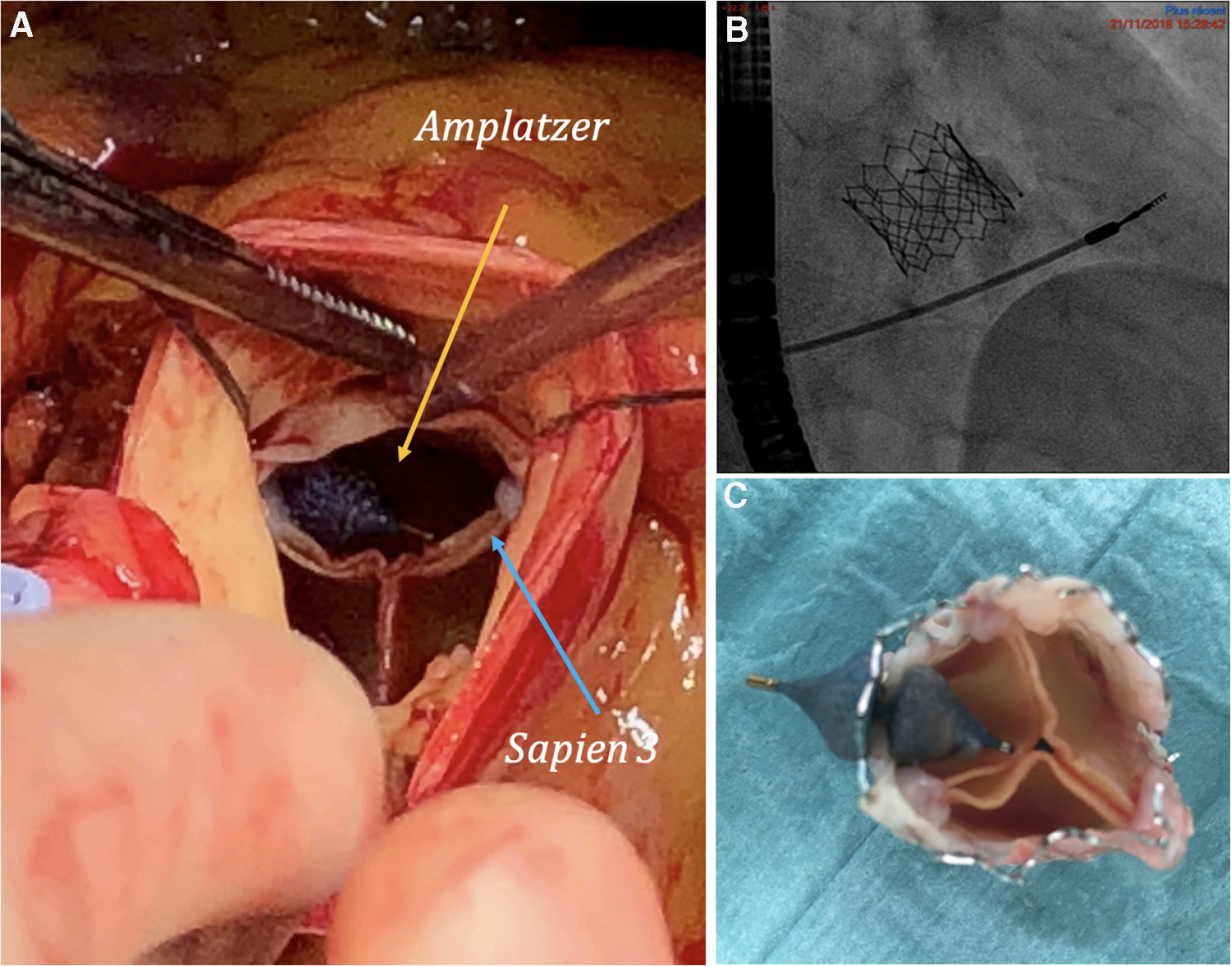
(**A**) Amplatzer vascular plug and bioprosthesis *in vivo*. (**B**) Angiography of initial implantation of Amplatzer vascular plug. (**C**) Bioprosthesis *ex vivo* with manual replacement of the Amplatzer vascular plug by the surgeon.

Given the new findings, the patient was referred for percutaneous repair of the paravalvular leak and was successfully implanted with an *Amplatzer Vascular Plug 4* of 8 mm (Figure 2B), resulting in a significant reduction of regurgitation and resolution of the heart murmur. Post-procedural echocardiography showed a residual paravalvular leak involving 1/5 of the valve perimeter, a calculated valve surface of 1 cm^2^, and a trans-aortic mean gradient of 14 mmHg.

**Figure 3:**
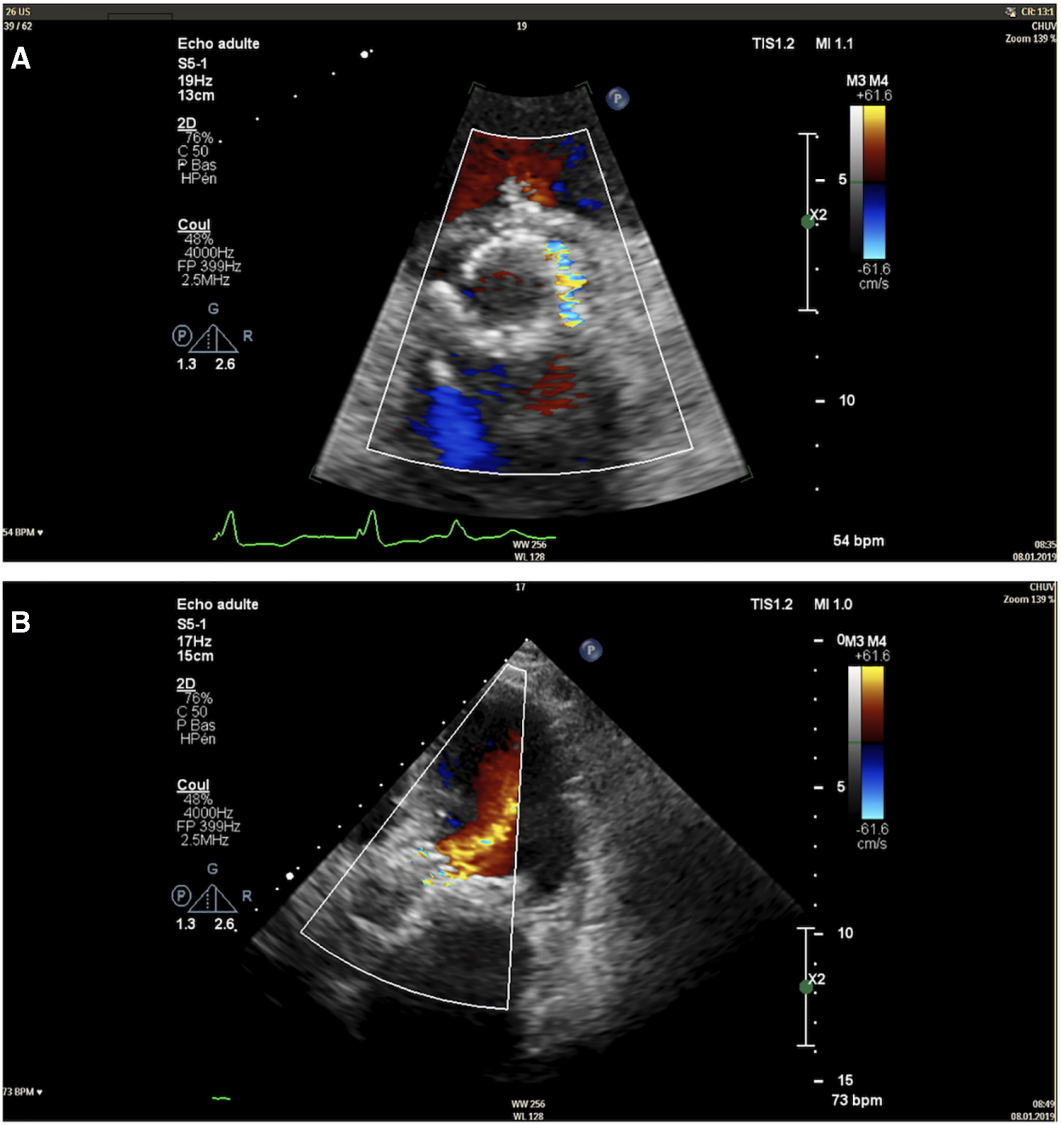
Transthoracic echocardiography image of the paravalvular leak in short-axis view (**A**) and apical five-chamber view (**B**).

Five weeks after the valvular leak repair, the patient was readmitted for acute heart failure. At this time, echocardiography showed mixed aortic disease with aortic stenosis characterised by a surface of 1 cm^2^, a mean gradient of 23 mmHg, and a significant paravalvular aortic regurgitation involving 1/3 of the valve perimeter (Figure 3). The case was reconsidered by the Heart Team, and a surgical approach was proposed.

During the surgical procedure, it was found that the *Amplatzer* had moved from its initial position and was oriented towards the leaflets of the bioprosthesis as shown in Figures 2 A,C. This contributed to the post-procedural aortic stenosis by impeding the opening of the right coronary leaflet and providing insufficient leak barrier. A surgical aortic valve replacement was performed using a sutureless LIVANOVA Perceval S bioprosthesis size M, which was completed without any complications. The postoperative echocardiography revealed a preserved left ventricle ejection fraction (62%) with grade 3 diastolic dysfunction, an aortic bioprosthesis with a calculated surface of 0.9 cm^2^, and a mean gradient of 18 mmHg with no regurgitation. The patient evolved well and was transferred to cardiac rehabilitation. The mid-term follow-up at 4 years showed a benefit of the procedures in terms of symptoms, with the patient now being in NYHA class I, and hospitalisations, with only one hospitalisation for acute heart failure since being discharged.

## Discussion

This case highlights two common complications of TAVI procedures and, more importantly, a complication of percutaneous paravalvular leak repair.

In recent years, new data have emerged regarding electrical conduction complications, when comparing surgical to percutaneous aortic valve replacement. For instance, the incidence of a new left bundle branch block is found to be 10.5%–34.3% in TAVI procedures (7), compared to 4% in surgical aortic valve replacements (8). Additionally, 7.3% of the patients undergoing percutaneous aortic valve replacement end up needing a permanent pacemaker implantation (all causes combined), compared to 3.4% in the surgical series (*p* = 0.014) (9). New left bundle branch block and new pacemaker implantation are some of the few outcomes that favour surgical approach.

With regards to the mechanical complication, this patient initially presented with a mild paravalvular leak that later progressed to a more significant one. The mechanism of this progression is unknown to date. Progression of paravalvular leaks has been described by the PARTNER trial (10); however, it happened over years instead of months seen in our case. So far, no mechanism has been proposed to explain the improvement or worsening of PVL, and measurement methods may explain, in part, these findings. The calcium volume described in the CT scan was low, and there were no indicators of preferential deposition in the device landing zone that could help predict this outcome.

Percutaneous valvular leak repair is a first-line treatment for paravalvular leaks and is nowadays associated with high success rates and few serious complications (11). However, it is a delicate and complex procedure and the correct choice of device and placement is essential. The literature concerning percutaneous paravalvular leak closure after TAVI is relatively scarce, and only a few case series have been published. The number of closures is too low to provide a good understanding of the complications. In contrast, more cases of complications after percutaneous PVL closure have been described in surgical series, and a few larger studies have been published in the literature that describe the most frequent complications encountered (Table 1).

**Table 1:** Literature review of case series of Percutaneous PVL closures and its complications.

First author	Year	PVL closures (*n*)	Type of procedure	Number and type of complications
Arri (12)	2015	5	TAVI	1 patient (20%) needed a second device implantation due to complexity of the leak
Gérardin (13)	2019	7	TAVI	No complications
Sorajja (14)	2011	154	Aortic 21%, mitral 79%	11 patients (8.7%) due to either inability to cross the defect, prosthetic leaflet impingement from the occluder, or persistent severe regurgitation
			Bioprosthesis 39%, mechanical 61%	
Ruiz (15)	2011	49	Aortic 22%, mitral 78%	8 patients (16%) due to inability to cross the defect with the delivery system, 3 patients (6%) due to device interference with the mechanical function of the valve prosthesis, and 1 patient (2%) due to wire entrapment during the attempt to cross the aortic paravalvular leak
			Bioprosthesis 65%, mechanical 35%	
Sorajja (16)	2011	115	Aortic 22%, mitral 78%	5 patients (4%) due to prosthetic leaflet impingement, 2 patients (1.7%) due to inability to cross with delivery sheath, 1 patient (0.9%) due to inability to cross with guidewire, 18 patients (15.6%) due to device deployed with residual moderate or severe regurgitation, and 1 patient (0.9%) due to malposition in left ventricle (LV)
			Bioprosthesis 37%, mechanical 63%	

TAVI, transcatheter aortic valve implantation.

In our patient's case, the Amplatzer device appears to have moved and intertwined with the metallic stenting of the prosthesis, which not only caused a failure in completely resolving the leak but also restricted the valve's full opening and led to a reduction in the valve surface, resulting in a new aortic stenosis. The reason for the device's migration is unknown, but it could be related to its initial position during the procedure.

These complications highlight the ongoing debate on indications between TAVI and surgical approaches. Despite the patient's vehement objection to a surgical approach, in light of her Euroscore/STS score and frailty score, and considering the small size of the valve annulus, a surgical approach may have yielded better results in this case. Ultimately, the patient underwent surgery anyway.

## Conclusion

To the best of our knowledge, this is the first case in which a TAVI paravalvular leak treated percutaneously with an *Amplatzer* device was complicated by the device's migration through the stenting of the bioprosthesis, resulting in a new symptomatic stenosis that required surgery. This highlights the need for better criteria for selecting the appropriate technique for managing paravalvular leaks after TAVI. Whether a baseline surgical approach or valve post-dilation would have been a better option in this patient remains unclear.

## Data Availability

The original contributions presented in the study are included in the article, further inquiries can be directed to the corresponding author.
